# Mental health service requirements after hospitalization due to COVID-19: a 1- year follow-up study

**DOI:** 10.1192/j.eurpsy.2022.966

**Published:** 2022-09-01

**Authors:** J. Andreo Jover, M.P. Vidal-Villegas, R. Mediavilla, I. Louzao Rojas, S. Cebolla Lorenzo, E. Fernández Jiménez, A. Muñoz-Sanjosé, M.F. Bravo-Ortiz, G. Martinez-Ales, C. Bayón-Pérez

**Affiliations:** 1 Autonomous University of Madrid, Department Of Psychiatry, Madrid, Spain; 2 Hospital La Paz Institute for Health Research (IdiPAZ), Psychiatry And Mental Health, Madrid, Spain; 3 Centro de Investigación Biomédica en Red de Salud Mental, Cibersam, Madrid, Spain; 4 La Paz University Hospital, Psychiatry, Clinical Psychology And Mental Health, Madrid, Spain; 5 Columbia University Mailman School of Public Health, Departament Of Epidemiology, Columbia, United States of America

**Keywords:** Hospitalized, 1-year Follow-up, Covid-19, psychiatry

## Abstract

**Introduction:**

Long-term COVID-19 effects has been recently described as persistent and prolonged symptoms after an acute and severe SARS-COV-2 (1). An important concern is that the sequelae of severe COVID-19 may suppose a substantial outpatient 's burden for the specialized services in reopening pandemic phase (2).

**Objectives:**

To describe the frequency of mental health service use in COVID-19 hospitalized patients after discharge and to estimate the costs associated to the post- discharge consultations.

**Methods:**

We used a 1-year follow-up cohort of 1455 COVID-19 inpatients hospitalized in La Paz University Hospital of Madrid, Spain between March 16th and April 15th, 2020. Data were retrieved from Psychiatry Service (PS) electronic health records and we described the frequency of mental health reason for consultation. We used information published by the Madrid health Office to estimate the cost of initial and following appointments.

**Results:**

Our sample consisted of 1,455 patients admitted with a COVID-19 diagnosis between March 16th and April 15th, 2020, and then discharged. Roughly half of them were men (776, 53%), 238 (16%) had a prior history of mental health problems, and 44 (3%) died. 193 participants (13%) visited the mental health department after being discharged. The total cost was estimated in 103,581 USD, of which two-thirds corresponded to patients with prior history of mental health problems.

**Conclusions:**

Our results indicate that the mental health burden of severe COVID-19 inpatient s after discharge was substantial during the first year of follow-up. This generate important economic impact to mental health providers and society at large.

**Disclosure:**

No significant relationships.

